# The Connection between Sleep Patterns and Mental Health: Insights from Rural Chinese Students

**DOI:** 10.3390/healthcare12151507

**Published:** 2024-07-30

**Authors:** Jiayang Lyu, Songqing Jin, Chen Ji, Ru Yan, Cindy Feng, Scott Rozelle, Huan Wang

**Affiliations:** 1 China Academy for Rural Development (CARD), Department of Agricultural Economics and Management, School of Public Affairs, Zhejiang University, 866 Yuhangtang Rd., Hangzhou 310058, China; 11922028@zju.edu.cn (J.L.);; 2 Stanford Center on China’s Economy and Institutions, Freeman Spogli Institute for International Studies, Stanford University, Stanford, CA 94305, USA; 3 Department of Agricultural, Food and Resource Economics, Michigan State University, 220 Trowbridge Rd., East Lansing, MI 48824, USA; 4 Tsingshan Institute for Advanced Business Studies, Zhejiang University, 866 Yuhangtang Rd., Hangzhou 310058, China

**Keywords:** sleep duration, bedtime, mental health, students, rural China

## Abstract

Background: The association between sleep patterns and young students’ mental health, which is crucial for their development, remains understudied in rural China. Therefore, the relationship between sleep patterns and mental health among primary and junior high school students in rural China was examined. Method: A total of 1592 primary and junior high school students from rural areas of Gansu Province were surveyed, and the Depression Anxiety and Stress Scale (DASS) was utilized to assess mental health, alongside self-reported data on their daily sleep patterns. Results: Significant sleep inadequacies were identified: 28% of students received less than 8 h of sleep on weekdays, and 19% went to bed later than recommended. On weekends, 38% of students had delayed bedtimes, though only 7.2% received less than 8 h of sleep. Notably, a “U-shaped” relationship was uncovered between sleep duration and mental health for students on weekends, with optimal mental health correlated with receiving 10–11 h of sleep, while both shorter and longer sleep durations on weekends worsened outcomes. This pattern is absent on weekdays. Additionally, adequate sleep and an earlier bedtime was linked to a 6–8% decrease in mental health risks. Conclusions: These findings provide valuable insights for policymakers seeking to enhance student mental well-being in rural settings, emphasizing the importance of implementing measures that promote balanced sleep habits among young students.

## 1. Introduction

Sleep patterns, encompassing sleep duration and wake-up/fall-asleep schedules, have been the subject of extensive research in the medical and psychological fields for many years. Insufficient sleep significantly affects both physical and mental health, raising the risk of heart disease and obesity [1,2,3,4]. It also increases the risk of depression and anxiety and raises stress levels [5,6,7,8]. On the other hand, adequate sleep duration has been associated with a reduction in mental illness symptoms [5,8].

The link between sleep duration and mental health is particularly critical among students. Evidence indicates that students are more susceptible to mental health issues due to insufficient sleep compared to the general population [7,9,10]. In high-income countries, there has been a concerning decrease in sleep duration among students over the past century, especially in Asian and North American countries, with a decline of nearly 1 h per night [11]. Insufficient sleep was found to be a common problem for 9–11-year-old fourth- and fifth-grade elementary students in America and Germany [12].

Previous sleep research has indicated that the relationship between sleep duration and mental health varies across age groups [13,14]. In general, students consistently fall short of the recommended 9 h of sleep on weekdays [15]. Yet younger students may be more vulnerable to insufficient sleep due to lower resilience and adjustment levels [16,17,18,19,20]. The growing prevalence of sleep problems among primary and junior high school students has become a concern [2,21,22]. The trend has been exacerbated by the COVID-19 pandemic, as lockdowns have led to increased time spent at home by young students, contributing to reduced sleep duration and delayed bedtimes [13]. This age group in particular therefore warrants increased attention. However, prior research has predominantly focused on high school and college students, with limited exploration into the sleep patterns of primary and junior high school students [23,24,25].

Moreover, studies indicating that students exhibit different sleep patterns on weekdays and weekends prompt the need to scrutinize the nuances of the relationship between sleep patterns and mental health. Prior research indicates that students often compensate for weekday sleep deficits by catching up on sleep over the weekend [26,27,28]. However, some researchers have detected a “U-shaped” relationship in the correlation coefficients between sleep patterns and mental health [29,30,31,32]. They found that both shorter (equal to or less than 7 h) and longer (equal to or more than 8.5 h) sleep durations throughout a week were significantly associated with depressive symptoms. Yet these findings lack confirmation through econometric models with quadratic terms for sleep duration, crucial for directly estimating this “U-shaped” relationship in regression analyses. Relying solely on correlation coefficients limits their capacity to thoroughly explore this relationship. To obtain more comprehensive insights, it is essential to employ empirical models considering other factors that could influence the association between sleep duration and mental health.

Despite the recent increase in international research on the link between student sleep patterns and mental health, a significant gap remains, particularly in understanding the sleep behaviors of primary and junior high school students in middle- and low-income countries. Current studies predominantly focus on urban areas and high-income nations [33,34,35,36]. While some researchers have explored how sleep patterns impact the mental health of students in China, their emphasis has been primarily on high school and college students [23,24,25]. The lack of evidence on the associations between sleep patterns and mental health among primary and junior high school students, particularly in rural settings, underscores the necessity to address the developmental disparities between urban and rural areas in China and prioritize the mental health of students in rural regions.

Rural areas in China, housing 40% of the population permanently and where 51% of children aged 0–17 receive schooling, exhibit significant developmental disparity with urban areas [37,38] (NBSC, 2016, 2019). Understanding the link between sleep patterns and mental health in primary and junior high school students in rural regions is thus crucial for several reasons. First, rural students often have lower academic performance than their urban counterparts [39,40]. Second, the significant development gap and limited resources available to rural students negatively impacts their development [41,42]. Third, primary and junior high school students in rural China face unique challenges, such as the absence of parental support due to out-migration for work, which significantly hinders their well-being and academic progress [43,44]. Above all, the limited research on this demographic underscores the need for further study to inform future studies and interventions [45,46,47,48].

Our study investigates the relationship between sleep and mental health among a rural population of primary and junior high school students in China. The primary objective is to examine the prevalence and correlates of mental health risks in relation to sleep patterns. This study aims to achieve three main objectives. First, we assess the prevalence of mental health risks among primary and junior high school students in rural China. Second, we investigate the sleep patterns of the sampled students, as well as the correlating factors affecting these patterns on both weekdays and weekends. Lastly, we delve into the relationship between sleep patterns—specifically, sleep duration and whether students stay up late—and mental health. The findings of our study will have practical implications, aiding in the design of targeted interventions and support systems to foster healthier sleep habits and bolster overall mental health for this vulnerable demographic.

## 2. Material and Methods

### 2.1. Ethical Approval

Ethical approval for this study was granted by the Stanford University Institutional Review Board (Protocol 58251). Written consent forms were sent to parents or guardians of eligible adolescents prior to conducting the survey. Throughout the study, we adhered to the Declaration of Helsinki to maintain data privacy and confidentiality. As such, adolescents were prohibited from discussing their responses during or after the survey, and we deleted the names of the sample adolescents from all electronic files during data encryption.

### 2.2. Data Collection

During October 2020, thirty schools were selected randomly from a list of all schools from the local education bureau in Gansu province, located in northwest China, which were needed to reach 80% statistical power based on power calculation. The selection comprised 20 primary schools and 10 junior high schools, ensuring a representative sample. For primary schools, we included fourth- and fifth-grade students, and for junior high schools we included seventh- and eighth-grade students. We excluded students in the sixth and ninth grades due to school entrance examination preparations, which could pose challenges in obtaining school approval for survey participation. We also excluded students in the first to third grades due to perceived age-related limitations. From each grade, we randomly chose two classes. Approximately half of the students in each selected class were then invited to partake in the survey. Our initial sample included 1609 students in 95 classes across the 30 sample schools. Due to missing data from 17 students, our final analytical sample consisted of 1592 students, giving a response rate of over 98%. A total of 1592 students from 95 sample classes voluntarily participated. Under the guidelines provided by the research team, students who participated in this survey were provided with survey forms and were asked to complete them under supervision to ensure reliability. All completed forms were collected at the end of the session by our research team. The survey included demographic questions and student’s daily time distributions and employed the Depression, Anxiety, Stress Scale (DASS) to assess students’ mental health.

### 2.3. Measurement of Variables

Student sleep patterns. Student sleep patterns in our study were assessed based on two main components: sleep duration and bedtime. In the demographic survey, students were asked to self-report the average time they went to sleep and woke up on both weekdays and weekends (our methodology involves the direct recording of sleep and wake times to the minute, which is a common method derived from validated sleep research instruments (like the Pittsburgh Sleep Quality Index (PSQI)). This approach is particularly suitable for younger students in rural China, enhancing data accuracy and ease of understanding due to its simplicity. A previous study demonstrated a significant correlation between self-reported sleep durations and those measured objectively among adolescents, underscoring the reliability and robustness of self-reported data [49]). To enhance the reliability of our data, we provided clear guidelines to respondents and their supervisors on how to record sleep times and encouraged consistency with daily routines in recording. From these data, we calculated the sleep duration for each student. To determine whether students obtained adequate sleep, we referred to the guidelines provided in the “Notice on Further Strengthening the Sleep Management of Primary and Middle School Students” issued by the General Office of the Ministry of Education. According to these guidelines, primary school students are advised to be asleep no later than 21:20, while junior high school students should be asleep no later than 22:00. Additionally, students between the ages of 6 and 13 are recommended to sleep for 9 to 11 h each night, and students between 14 and 17 are advised to sleep for 8 to 10 h. Using the recommended sleep duration and bedtime, we categorized our sample into two types of measurements: (1) adequate sleep on weekdays or weekends, represented as a dummy variable where “yes = 1” indicates meeting the recommended sleep duration and “no = 0” indicates otherwise; (2) late bedtime on weekdays or weekends, expressed as a dummy variable where “yes = 1” indicates going to bed later than the recommended time and “no = 0” indicates going to bed on time. In addition to the dummy variables, we also measured sleep duration using continuous variables. For sleep duration, we recorded the actual number of hours slept on both weekdays and weekends. As for bedtime, we opted to use only dummy variables. This decision was made to avoid the complexities associated with interpreting a continuous variable for bedtime (specifically, a continuous variable would imply a linear interpretation of bedtime hours (e.g., the shift from 9 p.m. to 10 p.m.), which does not straightforwardly translate into a meaningful economic or behavioral increment. Thus, we utilized a dummy variable to categorize students effectively without the ambiguity associated with continuous time points). By employing a combination of dummy and continuous variables, we obtained a comprehensive understanding of students’ sleep patterns, enabling us to explore the relationship between sleep and mental health more effectively in our study.

Student mental health. The Depression Anxiety and Stress Scale (DASS) is a well-established self-reporting instrument developed at the University of New South Wales to assess stress, depression, and anxiety. It consists of three separate scales, each comprising seven items (seven for depression, seven for anxiety, etc.). Participants rate each item on a scale from 0 (did not apply to me at all) to 3 (applied to me very much, or most of the time). The total score on the full-scale of the DASS ranges from 0 to 63, while the score for each dimension (Depression, Anxiety, and Stress) ranges from 0 to 21. The DASS is unique, as it pioneered the separate measurement of depression, anxiety, and stress. While these three dimensions have some overlap, the DASS clearly demarcates their differences. For this study, we utilized the Chinese version of the DASS-21, which was validated by [50] and is widely used in China and internationally as a reliable measure of mental health status.

In our study, we utilized dummy variables based on the standard DASS cutoff points to categorize mental health conditions into two groups: those without mental health problems and those with symptoms. We used the “moderate” symptom level as the baseline category for significant symptoms, assigning dummy variables a value of 1 for respondents whose scores exceed this threshold, indicating the presence of moderate or more severe mental health issues. Respondents scoring below this cutoff were assigned a value of 0, indicating no significant mental health problems. As a robustness check, we also applied the “mild” symptom cutoff for the definition of the mental health dummy. We chose to use dummy variables due to their clarity and ease of interpretation (specifically, the DASS score increments do not have equal clinical significance across the scale, making it challenging to interpret the implications of each point increase. However, transitioning across the threshold of a category clearly indicates an increase in the risk of having mental health problems). Additionally, to explore the potential non-linear relationship between sleep duration and mental health in greater detail, we also included continuous DASS scores in our analysis. While each incremental increase in a DASS score does not have a direct clinical interpretation, this method allowed us to capture broader trends in the data. By employing both dummy variables for initial categorical analysis and continuous variables for the analysis of more complex relationships, our study leveraged the strengths of both approaches: dummy variables provided clear, immediate insights into risk categories, while continuous variables offered a detailed examination of underlying patterns.

**Student resilience.** In our study, we opted to utilize the 25-item Connor-Davidson Resilience Scale (CD-RISC) as the primary tool for assessing the resilience levels of students. The CD-RISC is a well-established and widely recognized self-reporting instrument specifically designed to measure resilience [51]. Comprising 25 individual items, respondents are asked to evaluate each statement based on their personal experiences. To facilitate this, the scale employs a five-point Likert system that spans from 0 to 4. The points are defined as follows: “not true at all” is represented by a score of 0, “rarely true” is marked as 1, “sometimes true” corresponds to 2, “often true” is denoted by 3, and “true nearly all of the time” is given the highest score of 4. After all items are scored, the cumulative result can range from 0 to 100. Higher scores on the CD-RISC are indicative of greater resilience, signifying that the individual possesses a stronger capacity to bounce back from adversities. The CD-RISC has been shown to be reliable and has been validated among school-aged populations in China (Cronbach’s alpha coefficient = 0.89) [52]. The resilience scale was included as a control variable to account for individual differences in resilience that could potentially influence mental health outcomes independently of sleep patterns. This methodological choice was intended to strengthen the validity of our findings by mitigating the influence of potential confounding variables.

Student characteristics. Our analysis incorporated several covariates to account for students’ individual and household characteristics. These included age and gender (male or female) of the students. We also collected information on whether students were boarding at school (yes or no), whether they had rural hukou (yes or no), or whether they were the only children in the family (yes or no). To assess the education level of each student’s parents, we asked the students to report the highest education level for both parents, choosing from five categories: illiterate, primary school (six years of education), junior high school (nine years of education), high school (12 years of education), and college and above. We then created a dummy variable using high school education or above as a cutoff (>12 years of education, yes or no). Additionally, we identified students whose fathers or mothers had migrated for work for more than six months in the past year as “Migrant father” or “Migrant mother”, respectively. To gauge the household’s economic status, we documented ownership of seven selected items, such as electronics and vehicles, included in the National Household Income and Expenditure Survey, creating a family asset index. Students were also asked to report the time spent on screens/sports/reading (in minutes per day) as part of the data collection. Lastly, to control for students’ own abilities, we included their resilience scores and standardized math scores in the analysis. These covariates allow us to account for various factors that may influence the relationship between sleep patterns and mental health among students in rural China.

### 2.4. Statistical Analysis

Transitioning from data collection and measurement, our analysis consists of three primary parts. Firstly, we summarized the individual and household characteristics of the students in our sample, as well as their sleep patterns (including sleep duration, bedtime, and wake-up time) and their mental health status as measured by the Depression Anxiety and Stress Scale (DASS). Secondly, to assess whether sleep patterns varied based on student or household characteristics, we performed *t*-tests. Thirdly, we conducted an ordinary least square linear regression model to understand the relationship between students’ sleep patterns and their mental health. To control for potential confounding effects, all the covariates mentioned earlier, including students’ and families’ characteristics, were included in the regression model. Additionally, class fixed-effects (FEs) were incorporated to account for differences across classes. This approach of class FEs is essential for comparing the mental health of students in the same class but with different sleep durations or bedtimes. To address any potential issues related to clustering, the standard errors of all regressions were clustered at the school level.

All statistical analyses were performed using Stata 15.1. We considered *p*-values below 0.1 as statistically significant for our study. This comprehensive analysis allowed us to gain valuable insights into the relationship between sleep patterns and mental health among primary and junior high school students in rural China.

## 3. Results

### 3.1. Demographics

Table 1 presents a summary statistic of the sample students. On average, the students in our sample were 11.5 years old, with 55.2% being male and 44.8% female. A significant majority of the students had rural hukou (91.5%), while only a small proportion boarded at school (14.9%). Furthermore, the majority of students had siblings at home (86.3%). Regarding parental education, only 24.1% of fathers and 14.5% of mothers completed high school. More than half of the students’ fathers (57.6%) were away from home for more than six months in the past year, and a minor fraction of students (8.5%) had divorced parents. The average age of the students’ fathers was 41 years, while the average age of their mothers was 38 years. In our sample, students reported spending an average of 23.5 min on screen time each day, 26.8 min on reading time, and 13.3 min on sports activities. Additionally, the average resilience score for the students in our sample was 59.8.

### 3.2. Students’ Mental Health Status and Sleep Patterns

Table 2 provides a comprehensive overview of the students’ mental health and sleep patterns in our sample. Our findings indicate that depression, anxiety, and stress symptoms were prevalent among students in our sample. The proportion of students with abnormal mental health was significant, with 32.7% experiencing depression, 51.2% experiencing anxiety symptoms, and 28.7% experiencing stress symptoms. Among those students with abnormal mental health, we observed varying degrees of severity. For depression, 9.7% of students experienced mild depression, 14.3% experienced moderate depression, 5.1% experienced severe depression, and 3.6% experienced extremely severe depression. As for anxiety, 9.0% of the students reported mild anxiety symptoms, 18.7% reported moderate anxiety symptoms, 9.9% reported severe anxiety symptoms, and 13.6% reported extremely severe anxiety symptoms. Similarly, for stress, 11.4% of students reported mild stress symptoms, 10.8% reported moderate stress symptoms, 4.8% reported severe stress symptoms, and 1.7% reported extremely severe stress symptoms.

**Figure 1:**
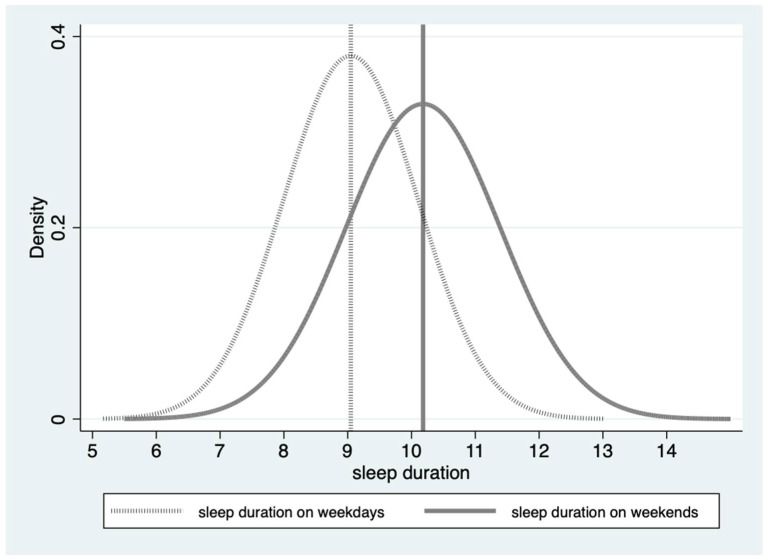
Distribution of students’ sleep duration on weekdays (mean = 9.05) and weekends (mean = 10.18). Notes: This figure illustrates the distribution of sleep duration between weekdays and weekends, with the dark solid line representing weekend sleep duration and the dotted line representing weekday sleep duration.

**Figure 2:**
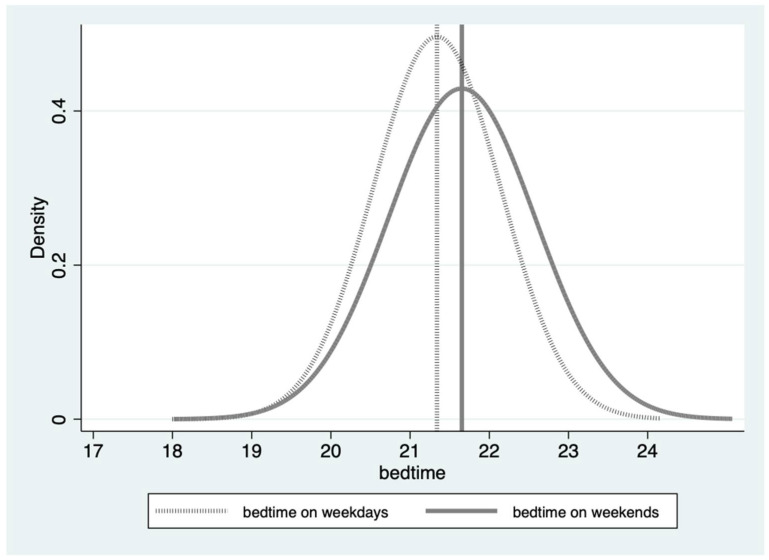
Distribution of students’ bedtime on weekdays (mean = 21:00) and weekends (mean = 21:30). Notes: This figure illustrates the distribution of bedtime between weekdays and weekends, with the dark solid line representing weekend bedtime and the dotted line representing weekday bedtime.

On average, students slept for 9 h on the weekdays and 10 h on the weekends, indicating that they were able to compensate for the lack of sleep during the week by sleeping more on weekends. Bedtime patterns showed that students typically went to bed at 21:30 on weekends and 21:00 on weekdays. Based on our standardized sleep metric, 28.3% of students did not get adequate sleep on a daily basis. Notably, the percentage of students reporting inadequate sleep was lower on weekends (7.2%) compared to weekdays. During weekdays, 19.3% of students struggled to fall asleep before the suggested bedtime, while 38% went to bed later than the recommended bedtime on weekends, which is approximately twice the rate as on weekdays. Some students even reported going to bed as late as 1:00 a.m. As displayed in Table 2, the majority of students in our sample managed to get adequate sleep on weekends; however, they tended to go to bed later. Figure 1 and Figure 2 illustrate the distribution of students’ sleep duration and bedtime on weekends and weekdays. The graphs reveal that bedtimes and sleep durations on weekdays were more concentrated and skewed to the left, indicating that bedtimes were earlier and sleep durations were shorter on weekdays compared to weekends. These findings shed light on the differences in sleep patterns between weekdays and weekends among primary and junior high school students in rural China.

1. The average bedtimes provided for weekdays and weekends (“Bedtime on weekdays, o’clock” and “Bedtime on weekends, o’clock”) are calculated by averaging the precise minute each respondent reported going to bed. This method ensures that the calculated bedtimes accurately represent the typical sleep patterns of the study population by summarizing individual responses.
2. This category includes any students who reported symptoms of depression. The mean value of 0.327 indicates that 32.7% of students reported varying levels of depression symptoms. These symptoms are further classified into mild, moderate, severe, and extremely severe categories, based on their severity levels. Percentages for mild (9.7%), moderate (14.3%), severe (5.1%), and extremely severe (3.6%) categories are calculated from the subset of students who reported experiencing depression symptoms, constituting 32.7% of the total sample. This breakdown helps to understand the distribution of symptom severity among students reporting depression. Similarly, this interpretation applies to the anxiety and stress scales of the DASS.

### 3.3. Correlating Factors of Sleep Patterns for Rural Students: Weekdays vs. Weekends

The *t*-test results presented in Table 3 reveal several findings regarding differences in sleep patterns among students while controlling for family characteristics. First, no significant differences were found in sleep patterns between male and female students. Second, older students exhibited lower probabilities of attaining sufficient sleep but were more inclined to go to bed earlier, particularly on weekdays. This implies that with age, students’ sleep patterns may be influenced by diverse factors contributing to decreased sleep duration and earlier bedtimes. These factors may include heightened academic demands, requiring students to go to bed early enough to ensure ample energy for the following school day yet making it more difficult for them to actually fall asleep (similar patterns regarding sleep duration among older students or those in higher grades have also been documented in other studies [32,53,54]). Third, students who boarded at school reported lower rates of obtaining sufficient sleep but higher rates of adhering to bedtime compared to their peers attending non-boarding schools. This discrepancy might be attributed to boarding students following school regulations and retiring to bed on time, although they may encounter challenges in falling asleep promptly, resulting in inconsistent sleep duration. Specifically, only 55.3% of boarding students achieved adequate sleep, in contrast to the 74.6% of non-boarding school students accomplishing this target. On average, non-boarding students tended to sleep later than boarding students, surpassing bedtime by 16.8 percentage points on weekdays and 25.2 percentage points on weekends. Fourth, students with longer screen time were more likely to experience insufficient sleep and go to bed later than the recommended time, particularly on weekdays. Students with longer screen time on weekdays were 10.8 percentage points less likely to get adequate sleep. On weekends, students with longer screen time were 3.9 percentage points less likely to get adequate sleep. Lastly, students whose parents had higher education levels were more likely to attain adequate sleep on weekdays, with 75–80% achieving sufficient sleep. However, a significant portion (26–56%) of these students still went to bed later than the recommended time.

### 3.4. Correlation between Students’ Sleep Patterns and Mental Health

#### 3.4.1. OLS Regression of Student Sleep Patterns and Mental Health

Table 4 presents the results of the multivariate OLS regression model, aiming to estimate the relationship between students’ sleep duration and their mental health. The dependent variable differs in columns (1)–(3) and (4)–(6). In columns (1)–(3), the dependent variable is whether the student had mild depression/anxiety/stress symptoms (coded as 1 for “yes”), while in columns (4)–(6), it is whether the student had moderate depression/anxiety/stress symptoms (coded as 1 for “yes”).

In our study, we observed a significant correlation between students’ sleep duration on weekdays and their depressive and anxiety symptoms. Adequate sleep duration was associated with a reduced probability of experiencing depression symptoms. Specifically, students who obtained adequate sleep on weekdays were at a lower risk for mild depression (6.1 percentage points, *p* < 0.1) and anxiety (7.4 percentage points, *p* < 0.05) compared to those with insufficient sleep on weekdays. Similar trends were evident for moderate severity of depression, anxiety, and stress symptoms (as shown in columns 4–6). A one-hour increase in sleep duration reduced the risk of mild depression symptoms by 4.9 percentage points (*p* < 0.01) and the risk of moderate depression symptoms by 6.0 percentage points (*p* < 0.01). We did not find any significant association between sleep duration on weekdays and stress symptoms. Furthermore, we did not find any relationship between sleep duration on weekends and mental health (depression, anxiety, and stress), regardless of how sleep duration was measured.

Table 5 presents the OLS regression results concerning whether students go to bed later than the recommended time and the association with mental health. The findings show that compared to students with earlier bedtimes, those who stay up later have a higher risk of experiencing mild depression, anxiety, and stress, as well as moderate depression and anxiety. Notably, this association is more significant on weekends, with larger and more statistically significant coefficients. On weekends, students with later bedtimes are more likely to suffer from mental illness and have a higher risk of experiencing mild depression (8.9 percentage points, *p* < 0.01), anxiety (8.4 percentage points, *p* < 0.01), and stress (9.5 percentage points, *p* < 0.01). Additionally, the risk of having moderate depression increases by 7.5 percentage points (*p* < 0.01), and the risk of moderate anxiety and stress also increases.

Overall, these findings indicate a notable association between later bedtime, particularly on weekends, and increased risks of mental health issues among young students. This underscores the significance of ensuring adequate sleep duration on weekdays and offers insights into the diverse impact of sleep duration and bedtime on young students’ mental health.

#### 3.4.2. The “U-Shaped” Relationship between Student Sleep Patterns and Mental Health

**Figure 3:**
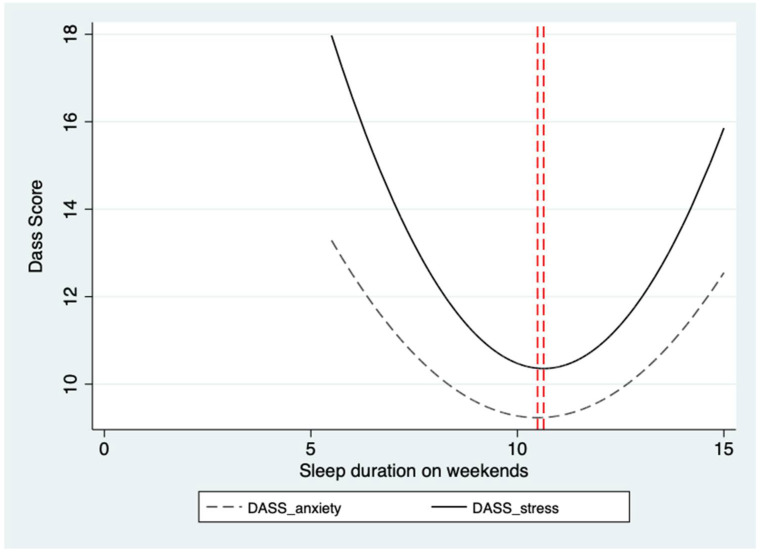
The “U-shaped” relationship between students’ sleep duration and mental health. Note: This figure presents the “U-shaped” relationship between students’ sleep duration on weekends and DASS total scores, illustrating that DASS total anxiety and stress scores decrease as the sleep duration increases; after reaching the extreme point, the score is increased again. The red dash lines are the mid-lines of the U-shaped curve, highlighting the symmetry of the graph.

Prior studies have identified a “U-shaped” relationship between sleep duration and mental health, indicating a negative correlation until reaching an optimal level, beyond which the risk of mental health issues increases with further increases in sleep duration [29,30,31,32]. In this study, we found a similar “U-shaped” relationship between sleep duration on weekends and students’ DASS total score (specifically in the anxiety and stress dimensions), as depicted in Figure 3. To examine this “U-shaped” relationship, we adjusted our regression model by including a quadratic term for sleep duration. We also incorporated class dummies in all regressions, and standard errors were clustered at the school level.

The results showed no “U-shaped” relationship between sleep duration on weekdays and mental health, signifying no significant correlation with mental problems, whether below or above the optimal level. However, for sleep duration on weekends, evidence of a “U-shaped” relationship with mental health emerged. Specifically, when sleep duration was below the optimal level, the risk of mental problems decreased with increasing sleep duration. Conversely, when sleep duration exceeded the ideal level, the relationship reversed, with mental health problems increasing as sleep duration increased. In Table 6, all regression models related to weekends passed the U-test, and the extreme point of the “U-shaped” relationship was observed to be around 11 h of sleep duration. This suggests that around 11 h of sleep on weekends may represent the optimal level for mental health, with increased risks of mental problems both for those who sleep significantly less or significantly more than this duration. These findings highlight the importance of considering the “U-shaped” relationship between sleep duration and mental health, particularly on weekends, to better understand how sleep patterns may correlate with students’ mental well-being.

## 4. Discussion

The current study explored the relationship between sleep patterns and mental health among primary and junior high school students. Using data from 1592 students across 30 schools in the impoverished rural areas of Gansu province, it reveals a prevalent trend of mental health concerns in this population, characterized by insufficient sleep and late bedtimes. Factors such as age, boarding status, screen time, and parental education levels significantly influence student sleep patterns, highlighting the complexity of sleep-related issues in this demographic. Furthermore, our findings suggest a negative relationship between inadequate sleep or late bedtimes and student mental health. Interestingly, excessive sleep is also detrimental to student mental health, evidenced by a “U-shaped” correlation found between students’ weekend sleep duration and their mental health outcomes. To the best of our knowledge, this study is the first to explore the relationship between sleep patterns and mental health in a younger population of rural Chinese students. Focusing on this age group is crucial, given their pivotal stage of learning and growth, where mental well-being has a profound influence on their future development.

### 4.1. The Relationship between Sleep Patterns and Mental Health in Young Students

First, our findings are consistent with prior studies [55,56] indicating that inadequate sleep (less than 8 h per night) and late bedtimes (after 22:00) predict increased depressive symptoms across various age groups. The consistency of the relationship across different demographics highlights the universal nature of inadequate sleep duration and late bedtime. Furthermore, similar results have been found in high-income countries or urban areas of China [57,58,59], demonstrating that the prevalence of these phenomena is not confined by socio-economic backgrounds or cultural differences. This further underscores the need for greater attention to the universal impact of sleep deprivation across different settings. Our study contributes to the existing knowledge by focusing on the relationship between sleep patterns and mental health relevance in primary and junior high school students, a group often neglected in sleep research. Educational programs aimed at teaching students and parents about the benefits of healthy sleep habits could prove highly advantageous. Additionally, schools should consider implementing policies that promote earlier bedtimes, such as limiting the amount of homework assigned and encouraging students to limit their screen time before bed [60,61,62]. Implementing these strategies could significantly improve sleep quality and, consequently, mental health outcomes in young students.

Second, our study did not reveal a significant linear relationship between sleep duration and symptoms of anxiety or stress. This finding contrasts with the literature, though we note that previous findings have been mixed. Some studies report associations between adequate sleep duration and anxiety [63,64], while others find that longer sleep duration might predict future anxiety symptoms [65]; some find no association at all [66]. These differing results may stem from interaction effects, reverse causality, or confounding factors [67]. According to our OLS regression analysis, the relationship between sleep duration and anxiety in our study is not statistically significant, hinting at a possible nonlinear association among these students. To explore this further, we included a detailed analysis using a quadratic term for sleep duration in our Ordinary Least Squares (OLS) regression models, as discussed in Section 4.2.

Third, our study uncovered differences in the relationship between student sleep patterns and mental health on weekdays versus weekends. We found that sufficient weekday sleep reduces depressive symptoms, while weekend sleep duration shows no clear correlation with mental health. Additionally, late bedtimes associate more strongly with depression, anxiety, and stress symptoms on weekends. This pattern may stem from early school start times and weekday homework pressures, leading to weekend “catch-up sleep” behavior. Weekend catch-up sleep has been found to be a common practice among school-aged children, with reported average durations of up to 3 h, including 1.17 h in the US and 1.5 h in China [68]. Moreover, previous studies have attributed reduced sleep on weekdays (leading to a higher instance of weekend catch-up sleep) to pubertal onset and growing academic demands on younger students [69]. Our findings, consistent with other research [68,70,71], emphasize the need to consider both weekdays and weekends when exploring sleep patterns and mental health in primary and junior high students. Because balancing sleep routines throughout the week is crucial for student mental well-being, further investigation into weekend catch-up sleep behavior is warranted. Schools and parents should prioritize helping students maintain an ideal sleep duration during weekdays.

### 4.2. The “U-Shaped” Relationship on Weekends

Our study identifies a significant “U-shaped” correlation between weekend sleep duration and mental health among primary and junior high school students, aligning with prior research that links both insufficient and excessive weekend sleep to higher depression and anxiety risks [57]. To delve deeper into this relationship, we introduced a quadratic term for sleep duration in our model, and the results revealed a “U-shaped” correlation between sleep duration on weekends and mental health. This finding is consistent with similar studies conducted across diverse age groups and populations. For instance, the relationship between sleep duration and anxiety has also exhibited a “U-shaped” pattern [72,73]. Furthermore, a study sampling Chinese adolescents using longitudinal data found a “U-shaped” association between sleep duration on weekdays, on weekends, or the difference between the two, and mental health [32].

In our sample, we observed that the “U-shaped” relationship was especially pronounced on weekends. This trend could be attributed to the students’ tendency to oversleep on weekends due to weekday school schedules and limited sleep opportunities [74]. Our findings emphasize the importance of raising awareness among parents and educators about the potential mental health implications of varying sleep patterns, depending on the day of the week. This is especially relevant given the high proportion of migrant parents in our sample (approximately 57% of fathers and 25% of mothers), who may encounter challenges in monitoring their children’s sleep habits. Recognizing that sleep patterns are significantly shaped within the home environment, addressing this lack of oversight becomes crucial [75,76].

Early identification and targeted intervention are essential to mitigate the negative impacts of poor sleep habits on students’ mental health. Our findings not only shed light on the complex relationship between sleep patterns and mental health among rural students in China but also underscore the importance of parental and educational awareness in supporting students’ well-being, particularly in the face of varied sleep patterns and the unique challenges migrant parents face.

### 4.3. Limitations

The study has several limitations. Firstly, the cross-sectional nature of our study restricted our capacity to infer causality between sleep patterns and mental health outcomes. This limitation is particularly significant in understanding the potentially bidirectional relationship between these variables, as changes in sleep patterns could affect mental health, and conversely, mental health issues might influence sleep patterns. Future longitudinal studies could provide deeper insights into how these relationships develop or change over time, aiding in establishing clearer causal linkages and understanding the dynamic interplay between sleep and mental health. Secondly, our assessment of sleep patterns and mental health was based on self-reported data. We documented students’ bedtime and wake-up times on weekdays and weekends, rather than employing established scales such as the Pittsburgh Sleep Quality Index (PSQI) or the Athens Insomnia Scale (AIS), which might have provided more accurate measures of sleep patterns and quality [77,78,79]. For economic, technological, or practical reasons, we did not employ apps on smartphones or wearables that could have offered more objective and specific records of sleep patterns. Utilizing these comprehensive and validated scales could potentially enhance the accuracy and reliability of our findings. Thirdly, the data were exclusively collected from Gansu Province in China, possibly limiting the generalizability of our findings to other regions. Data collection was confined to Gansu Province, which may not represent other regions, particularly urban or high-income areas in China. This limitation restricts the generalizability of our findings across different cultural, socioeconomic, and environmental contexts. Despite these limitations, our study highlights the importance of maintaining a regular sleep schedule and closely monitoring adolescents’ mental health. Given our findings, we recommend that governments and educational institutions implement policies to ensure regular sleep durations and adherence to recommended bedtimes for students.

## 5. Conclusions

Analyzing data from October 2020 in primary and junior high schools, we conducted regression analysis to explore the relationship between student sleep patterns and mental health. Firstly, there is a notable discrepancy in sleep patterns between weekdays and weekends, with a higher proportion of students obtaining adequate sleep during the latter. Secondly, within our sample, sleep patterns displayed variations based on individual and family characteristics, such as age, whether boarding at school, screen time, and parents’ education levels. Lastly, the relationship between sleep patterns and mental health exhibits distinctions between weekdays and weekends, highlighting the crucial role of both sleep duration and bedtime in influencing mental well-being. These insights contribute to a deeper understanding of the complex interplay between sleep and mental health among primary and junior high school students. Notably, extending sleep on weekdays reduces the risk of mental health issues, emphasizing the need for proper sleep duration to mitigate mental health risks. This study contributes to understanding the connection between sleep patterns and mental health, offering insights for performance improvement and strategies for schools and families to enhance sleep quality and overall well-being.

## Figures and Tables

**Table 1:** Summary of individual and family characteristics of students.

Variable	Obs.	Mean	Std.Dev.	Min.	Max.
Individual Characteristics					
Age, years	1592	11.535	1.618	7.833	15.583
Female, 1 = yes	1592	0.448	0.497	0	1
Board at school, 1 = yes	1592	0.149	0.357	0	1
Only child, 1 = yes	1592	0.137	0.344	0	1
Rural hukou, 1 = yes	1592	0.915	0.280	0	1
Family Characteristics					
Migrant father, 1 = yes	1592	0.576	0.494	0	1
Migrant mother, 1 = yes	1592	0.253	0.435	0	1
Father’s education level, 1 = high school or above	1592	0.241	0.428	0	1
Mother’s education level, 1 = high school or above	1592	0.145	0.352	0	1
Father age, years	1592	41.094	6.149	23	71
Mother age, years	1592	38.279	5.769	21	66
Divorced parents, 1 = yes	1592	0.085	0.279	0	1
Family asset index	1590	−0.015	1.239	−2.241	2.909
Time Usage on Extracurricular activities					
Screen time, minutes	1591	23.578	32.547	0	180
Sport time, minutes	1591	13.301	21.504	0	130
Read time, minutes	1591	26.792	19.031	0	120
Students Abilities					
Resilience (Total CD-RISC scores)	1592	59.872	14.139	0	100
Standardized math test score	1591	0.012 ^1^	0.959	−4.016	1.902

Notes: Table 1 presents the summary statistics of the students in our sample. Because of the missing values in our sample, the mean standardized math test score was not exactly equal to zero after we dropped a few observations. ^1^ Due to missing values in our sample, the mean standardized math test score is not exactly zero after we dropped several observations.

**Table 2:** Sleep patterns and mental health status of students.

Variable	Obs.	Mean	Std.Dev.	Min.	Max.
Sleep Patterns					
Sleep duration on weekdays, hours	1589	9.049	1.050	5.170	13.000
Sleep duration on weekends, hours	1565	10.181	1.211	5.500	15.000
Gain adequate sleep on weekdays, 1 = yes	1589	0.717	0.450	0	1
Gain adequate sleep on weekends, 1 = yes	1565	0.928	0.258	0	1
Bedtime on weekdays, o’clock ^1^	1591	21:00	0.803	18:00	24:00
Bedtime on weekends, o’clock	1590	21:30	0.929	18:00	1:00
Sleep late on weekdays, 1 = yes	1589	0.193	0.395	0	1
Sleep late on weekends, 1 = yes	1571	0.380	0.486	0	1
Student Mental Health Risks					
Depression (1 = yes) ^2^	1592	0.327	0.469	0	1
no depression symptoms	1592	0.673			
mild	1592	0.097			
moderate	1592	0.143			
severe	1592	0.051			
extremely severe	1592	0.036			
Anxiety (1 = yes)	1592	0.512	0.500	0	1
no anxiety symptoms	1592	0.488			
mild	1592	0.090			
moderate	1592	0.187			
severe	1592	0.099			
extremely severe	1592	0.136			
Stress (1 = yes)	1592	0.287	0.453	0	1
no stress symptoms	1592	0.713			
mild	1592	0.114			
moderate	1592	0.108			
severe	1592	0.048			
extremely severe	1592	0.017			

Notes: Table 2 includes sleep patterns, which were collected by asking students about their weekday and weekend waking up and going-to-sleep times. Additionally, mental health was measured using the DASS-21 scale, which consists of 21 items. The mean scores obtained on the DASS-21 by the total sample are 7.621 (depression), 9.487 (anxiety), and 10.822 (stress).

**Table 3:** The distribution of students’ sleep patterns by demographic characteristics.

	(1)		(2)		(3)		(4)	
Demographic Characteristics	Gain Adequate Sleep on Weekdays, 1 = Yes		Gain Adequate Sleep on Weekends, 1 = Yes		Go to Sleep Late on Weekdays, 1 = Yes		Go to Sleep Late on Weekends, 1 = Yes	
	Mean	Diff.	Mean	Diff.	Mean	Diff.	Mean	Diff.
Individual Characteristics								
Gender								
Female	0.721	0.006	0.957	0.052 ***	0.196	0.006	0.365	−0.028
Male	0.715		0.905		0.191		0.392	
Age								
≥12	0.458	−0.439 ***	0.907	−0.037 ***	0.077	−0.196 ***	0.190	−0.322 ***
<12	0.897		0.944		0.273		0.512	
Board at School								
Yes	0.553	−0.194 ***	0.910	−0.021	0.051	−0.168 ***	0.166	−0.252 ***
No	0.746		0.932		0.218		0.418	
Extracurricular activities								
Screen Time Exceeds 1 h/Day								
Yes	0.625	−0.108 ***	0.895	−0.039 **	0.254	0.071 **	0.504	0.146 ***
No	0.733		0.934		0.183		0.359	
Family Characteristics								
Migrant Father								
Yes	0.717	0	0.928	−0.002	0.187	−0.015	0.390	0.025
No	0.718		0.929		0.202		0.366	
Migrant Mother								
Yes	0.728	0.014	0.931	0.004	0.170	−0.032	0.367	−0.017
No	0.714		0.927		0.201		0.384	
Father’s Education > 9 Years								
Yes	0.755	0.049	0.936	0.009	0.261	0.089 ***	0.481	0.133 ***
No	0.706		0.926		0.172		0.348	
Mother’s Education > 9 Years								
Yes	0.801	0.098 ***	0.928	0	0.277	0.098 ***	0.566	0.218 ***
No	0.703		0.928		0.179		0.349	

Note: The distributions in Table 3 are the proportions of students who gain adequate sleep or have a late bedtime across different individual or family characteristics. *** *p* < 0.01, ** *p* < 0.05.

**Table 4:** OLS regression of students’ sleep duration and mental health.

	(1)	(2)	(3)	(4)	(5)	(6)
	DASS Mild Depression	DASS Mild Anxiety	DASS Mild Stress	DASS Moderate Depression	DASS Moderate Anxiety	DASS Moderate Stress
Adequate Sleep	−0.061 *	−0.074 **	−0.036	−0.088 ***	−0.062 *	−0.023
(Weekdays)	(−1.974)	(−2.131)	(−0.996)	(−3.304)	(−1.960)	(−1.080)
Control Variables	Y	Y	Y	Y	Y	Y
Class FE	Y	Y	Y	Y	Y	Y
Observations	1586	1586	1586	1586	1586	1586
adj. R^2^	0.108	0.069	0.038	0.111	0.095	0.029
Adequate Sleep	0.006	−0.047	−0.011	−0.040	−0.049	−0.024
(Weekends)	(0.111)	(−0.764)	(−0.237)	(−0.932)	(−0.810)	(−0.499)
						
Control Variables	Y	Y	Y	Y	Y	Y
Class FE	Y	Y	Y	Y	Y	Y
Observations	1563	1563	1563	1563	1563	1563
adj. R^2^	0.105	0.068	0.036	0.105	0.091	0.031
Sleep Duration	−0.049 ***	−0.038	−0.015	−0.060 ***	−0.040 *	−0.015
(Weekdays)	(−3.341)	(−1.526)	(−0.741)	(−4.910)	(−1.716)	(−1.083)
						
Control Variables	Y	Y	Y	Y	Y	Y
Class FE	Y	Y	Y	Y	Y	Y
Observations	1586	1586	1586	1586	1586	1586
adj. R^2^	0.110	0.069	0.037	0.115	0.096	0.030
Sleep Duration	−0.008	−0.001	−0.005	−0.010	−0.003	−0.004
(Weekends)	(−0.814)	(−0.056)	(−0.478)	(−1.099)	(−0.251)	(−0.419)
						
Control Variables	Y	Y	Y	Y	Y	Y
Class FE	Y	Y	Y	Y	Y	Y
Observations	1563	1563	1563	1563	1563	1563
adj. R^2^	0.106	0.067	0.037	0.105	0.091	0.031

Notes: The dependent variables are mild mental health symptoms in columns (1)–(3) and moderate mental health symptoms in columns (4)–(6). We employed two methods to measure sleep duration: continuous and dummy variables. Additionally, we recorded students’ sleep duration on both weekdays and weekends to study the differences. All regressions include dummies for class, and standard errors are clustered at the school level. T-statistics are reported in parentheses. *** *p* < 0.01, ** *p* < 0.05, * *p* < 0.1.

**Table 5:** OLS regression of students’ going to sleep late and mental health.

	(1)	(2)	(3)	(4)	(5)	(6)
	DASS Mild Depression	DASS Mild Anxiety	DASS Mild Stress	DASS Moderate Depression	DASS Moderate Anxiety	DASS Moderate Stress
Going to Sleep Late	0.049 **	0.046	0.061 *	0.074 ***	0.069 ***	0.027
(Weekdays)	(2.354)	(1.684)	(2.040)	(3.229)	(2.953)	(1.030)
Control Variables	Y	Y	Y	Y	Y	Y
Class FE	Y	Y	Y	Y	Y	Y
Observations	1586	1586	1586	1586	1586	1586
adj. R^2^	0.107	0.067	0.039	0.110	0.096	0.030
Going to Sleep Late	0.089 ***	0.084 ***	0.095 ***	0.075 ***	0.104 ***	0.055 **
(Weekends)	(3.236)	(3.338)	(3.709)	(2.871)	(4.491)	(2.597)
Control Variables	Y	Y	Y	Y	Y	Y
Class FE	Y	Y	Y	Y	Y	Y
Observations	1569	1569	1569	1569	1569	1569
adj. R^2^	0.113	0.073	0.046	0.111	0.100	0.033

Notes: The dependent variables are mild mental health symptoms in columns (1)–(3) and moderate mental health symptoms in columns (4)–(6). The dependent variable is a dummy variable about whether students stay up late or not on weekdays or weekends to measure bedtime. Additionally, to study the difference in bedtime between weekdays and weekends, we recorded the students’ bedtime separately for weekdays and weekends. All regressions include dummies for class, and standard errors are clustered at the school level. *T*-statistics are reported in parentheses. *** *p* < 0.01, ** *p* < 0.05, * *p* < 0.1.

**Table 6:** The “U-shaped” relationship between students’ sleep duration and mental health.

	(1)	(2)	(3)	(4)	(5)	(6)
	DASS Score (Depression)		DASS Score (Anxiety)		DASS Score (Stress)	
Sleep duration	−2.823		−1.314		−3.958	
(Weekdays)	(−1.067)		(−0.560)		(−1.478)	
Sleep duration squared	0.111		0.035		0.188	
(Weekdays)	(0.731)		(0.265)		(1.214)	
Sleep duration		−3.120		−3.863 **		−5.896 ***
(Weekend)		(−1.408)		(−2.353)		(−3.174)
Sleep duration squared		0.142		0.175 **		0.276 ***
(Weekend)		(1.382)		(2.256)		(3.116)
U-test (*p*-value)	0.484	0.109	-	0.030	0.253	0.004
Extreme point	12.752	10.982	-	11.016	10.520	10.689
Control Variables	Y	Y	Y	Y	Y	Y
Class FE	Y	Y	Y	Y	Y	Y
Observations	1586	1563	1586	1563	1586	1563
adj. R^2^	0.119	0.118	0.062	0.061	0.043	0.045

Notes: The dependent variables are DASS scores in columns (1)–(6). We included quadratic terms in our model to estimate the ‘U-shaped’ relationship between sleep duration and mental health. All regressions include dummies for class, and standard errors are clustered at the school level. T-statistics are reported in parentheses. *** *p* < 0.01, ** *p* < 0.05.

## Data Availability

The data presented in this study are available on request from the corresponding author. The data are not publicly available, as they contain sensitive information on the mental health of children and adolescents in rural China.
